# Missed opportunities for hypertension screening of older people in the Philippines: cross-sectional analysis of nationally representative individual-level data

**DOI:** 10.1016/j.lanwpc.2024.101188

**Published:** 2024-09-04

**Authors:** Aleli D. Kraft, Joseph J. Capuno, Kayleen Gene R. Calicdan, Grace T. Cruz, Owen O'Donnell

**Affiliations:** a School of Economics, University of the Philippines Diliman, Philippines; b Population Institute, University of the Philippines Diliman, Philippines; c School of Health Policy & Management, School of Economics, Erasmus University Rotterdam, the Netherlands

**Keywords:** Hypertension, Blood pressure, Screening, Diagnosis, Cardiovascular diseases, Inequality, Philippines

## Abstract

**Background:**

Guidelines recommend routine blood pressure measurement at health facilities. We estimated the potential for opportunistic screening for hypertension at health facilities to change the level and distribution of diagnosed hypertension in the older population of the Philippines.

**Methods:**

We used a representative, nationwide sample of Filipinos aged 60 years and older and classified respondents as a) *hypertensive* if they had high (≥140/90 mm Hg) blood pressure (BP) or were taking BP medication, b) *diagnosed* if told have high BP by a doctor, and c) a *missed opportunity for diagnosis* if they were hypertensive, undiagnosed and had an outpatient visit to a health facility in the past 12 months. We assumed c) would be diagnosed if health facilities operated opportunistic screening. We estimated percentages of hypertensives diagnosed and with a missed opportunity overall, by wealth quintile and covariates, with age-sex and, then, full adjustment.

**Findings:**

We estimated that opportunistic screening at health facilities would increase the percentage of hypertensives diagnosed from 62.7% (95% CI: 58.2, 67.0) to 74.4% (95% CI: 70.9, 77.6). The increase would be larger in richer groups due to lower (private) healthcare utilization by poorer, undiagnosed hypertensives.

**Interpretation:**

Opportunistic screening for hypertension, if effectively implemented at health facilities, would substantially increase diagnosis but exacerbate inequality unless barriers discouraging poorer, older Filipinos from accessing outpatient and primary care were lowered.

**Funding:**

10.13039/100020917Economic Research Institute for ASEAN and East Asia, 10.13039/100009131Swiss Agency for Development and Cooperation/10.13039/501100001711Swiss National Science Foundation grant 400640_160374.

**Research in context**

**Evidence before this study**

We searched on PubMed and Google Scholar for articles on hypertension diagnosis and opportunistic screening for hypertension in low- and middle-income countries (LMICs), with particular attention to the Western Pacific, older adults and socioeconomic inequality. We used search terms including “diagnosis” OR “care cascade” OR “opportunistic screening” OR “elderly” OR “older persons” OR “low- and middle-income countries” OR “Western Pacific” OR “the Philippines” OR “inequality” AND “hypertension”. We screened the resulting articles to identify those with evidence on hypertension diagnosis or opportunistic screening in LMICs, and focused on those including older populations.

Many studies documented high prevalence and low diagnosis of hypertension in older populations of LMICs. Hypertension care cascade studies established that underdiagnosis of hypertension contributes substantially to low control of blood pressure. Underdiagnosis of hypertension tends to be higher in socially disadvantaged groups.

There is some evidence on opportunistic screening for hypertension from detailed, local studies but very little evidence from nationally representative data. Studies undertaken using such data from India, Sri Lanka and Mexico demonstrated that opportunistic screening at health facilities has the potential to substantially increase the diagnosis of hypertension without worsening socioeconomic inequality in diagnosis. Evidence on hypertension diagnosis and screening in the Western Pacific region is mostly from China.

**Added value of this study**

To our knowledge, this is the first study to use nationally representative individual-level data from a Western Pacific country to estimate the potential for opportunistic screening for hypertension at health facilities to increase diagnosis of the condition and change socioeconomic inequality in the diagnosis rate among older persons. By estimating impacts by sociodemographic characteristics and distinguishing between missed opportunities for screening at public and private health facilities, we were able to identify gaps in hypertension diagnosis that opportunistic screening could help close and others that it may widen.

The study demonstrated that opportunistically screening older Filipinos for hypertension at health facilities could substantially increase diagnosis, on average, but would likely exacerbate socioeconomic inequality in underdiagnosis. It would only marginally reduce the greater underdiagnosis of males compared with females.

**Implications of all the available evidence**

Effective implementation of opportunistic screening for hypertension at health facilities can increase diagnosis of a condition that is the number one risk factor for attributable deaths worldwide despite its low cost of detection and management with effective medication. Substantial challenges to effective implementation include freeing up capacity of under-resourced health facilities to screen for a highly prevalent condition. Implementation at highly utilized private health facilities is a particular challenge. The distributional impact of opportunistic screening will reflect inequalities in healthcare utilization. Increasing diagnosis of hypertension in hard-to-reach poor, rural populations may require either interventions to lower barriers to access alongside opportunistic screening or complementary targeted population screening.

## Introduction

In 2019, high systolic blood pressure was the global leading Level 2 risk factor for attributable deaths^1^ and accounted for the largest attributable burden of cardiovascular diseases (CVDs).^2^ Worldwide, these diseases continue to be the leading cause of death^3^ and the second to top contributor to the global burden of disease.^4^ Around four fifths of CVD deaths occur in low- and middle-income countries (LMICs), where the decline in CVD mortality rates is much slower than in high-income countries.^3^^,^^5^

While global hypertension prevalence has been stable over the last three decades, prevalence has continued to rise in the Western Pacific.^6^^,^^7^ That region has the largest number of people living with hypertension and has contributed more than any other to the global hypertensive population doubling since 1990.^6^^,^^7^ Compared with other World Health Organization (WHO) regions, the Western Pacific also accounts for most deaths attributable to high systolic blood pressure and had the largest increase in those deaths in the last thirty years.^6^^,^^7^ Most countries in the region are not on track to reach SDG target 3.4—a one third reduction mortality attributable to the four leading noncommunicable diseases (NCDs) by 2030.^8^

Central conduit artery stiffness, associated with ageing, contributes to higher prevalence of hypertension at older ages.^9^ Population ageing is expected to further increase hypertension prevalence in Asian LMICs.^10^ However, clinical trials demonstrate effective treatments for hypertension at older ages that offer potential for management of the condition to reduce CVD risks in older populations.^9^ Realization of this potential hinges on timely diagnosis.^11^

Hypertension is largely asymptomatic, often being diagnosed only after it has damaged vital organs.^12^, ^13^, ^14^ There is substantial underdiagnosis of hypertension in LMICs^6^^,^^15^, ^16^, ^17^ and in the Western Pacific.^18^^,^^19^ While hypertension prevalence does not have a clear association with socioeconomic status in LMICs,^20^ underdiagnosis of the condition tends to be even higher among the socially and economically disadvantaged.^16^^,^^17^^,^^21^

The WHO Package of Essential Noncommunicable Disease Interventions for Primary Healthcare (PEN) recommends routine blood pressure measurement of patients on presentation at any health facility for any reason.^22^, ^23^, ^24^ The Philippines’ version of the PEN protocol stipulates that all patients aged 25 years and older presenting at public health clinics should undergo CVD risk assessment, including blood pressure measurement.^25^ Those with raised blood pressure (or aged ≥40 years) should be screened for CVD risk, and those with high blood pressure should be prescribed antihypertensives that public clinics are obliged to provide without charge.^25^^,^^26^ Notwithstanding these policies, there is evidence that opportunistic screening for hypertension does not operate systematically even at public clinics.^14^^,^^27^

This study aimed to quantify the extent to which effective implementation of opportunistic screening at all health facilities in the Philippines would increase the diagnosis of hypertension and change inequality in the rate of diagnosis in the older population of a country where the health and economic burden of the condition is projected to remain high.^28^

## Methods

### Data

We used data from the baseline of the Longitudinal Study of Ageing and Health in the Philippines (LSAHP), conducted from October 2018 to February 2019.^29^ Multistage sampling was used to obtain a nationally representative sample of Filipinos aged 60 years and older (60+).^30^ Provinces, and municipalities in the National Capital Region, were stratified based on the proportion of older persons (OPs) obtained from 2018 population projections. Within each of three strata, provinces/municipalities were selected using systematic sampling. In each of the selected nine provinces and two municipalities, barangays (villages/neighborhoods) were selected by probability-proportional-to-size sampling, with size defined by the population aged 60+, and by implicit urban/rural stratification. For each of the 167 sampled barangays, the LSAHP study team compiled a comprehensive list of all resident OPs, that served as the sampling frame for random selection. Out of a target sample of 6335 OPs invited through home visits, complete interviews were conducted with 5985 (94%) consenting OPs or proxy respondents.^29^

### Measurements and outcomes

Blood pressure (BP) was measured three times at 1-min intervals using an Omron HEM-7120 digital monitor. BP was not measured if the respondent did not give consent or a monitor could not be fitted on an arm. Occasionally, less than three measurements were taken because of monitor error or withdrawal of consent. We used the average of the last two measurements. A respondent was classified as *hypertensive* if a) they had systolic BP ≥ 140 mm Hg or diastolic BP ≥ 90 mm Hg or b) they reported currently taking medication for high blood pressure.^6^^,^^7^^,^^31^ A respondent was classified as *diagnosed* if they reported having been told by a doctor that they have high blood pressure.

Each respondent was asked: *In the past* 12 months*, have you received medical care for an illness or accident from any medical facility or practitioner without staying overnight?* We classified a respondent as having a *missed opportunity* for hypertension diagnosis if i) they answered *YES* to this question, ii) they met the criteria for being classified as *hypertensive,* and iii) they did not meet the criterion for being *diagnosed.*^32^^,^^33^ We classified a respondent as *potentially diagnosed* if they were classified either as *diagnosed* or as a *missed opportunity*.

If a respondent reported having received medical care in the past 12 months, they were asked about the type of health facility visited. We distinguished missed opportunities at public facilities (barangay health stations, rural health units, and municipal/community, district, city, provincial, regional, national and specialty hospitals) from those at private facilities (private clinics or hospitals or those not classified as either public or private). We restricted attention to health facility visits without an overnight stay in order to estimate the potential impact of opportunistic screening at outpatient clinics and in primary care.

### Covariates

We applied principal component analysis to indicators of household ownership of 14 durable assets, house ownership/tenure, electricity supply, internet access, water supply, sanitation, housing materials and receipt of a cash transfer targeted at the poor (Supplementary Table S1). We used the first principal component as a wealth index and its rank as a proxy for economic status.^34^^,^^35^ We used the index to categorize respondents into wealth quintile groups. To maintain comparability between urban and rural residents, we used the same index for both. Other covariates were reported sex (male, female), age categorized into 5-year groups (60–64 years, 65–69 years, …,80+ years), location (rural, urban), highest educational attainment (elementary school or less, high school or vocational, college), living arrangement (alone, with spouse only, with children, other), employment (working, not working), health insurance (yes, no) and indicators of province/municipality.

### Statistical analysis

We restricted the analysis sample to respondents with three BP measurements and used it to estimate hypertension prevalence (the percentage classified as hypertensive) in the older population and by wealth group and other covariates (adjusted for age and sex). There were no missing data on wealth and covariates. We restricted this sample to those classified as hypertensive and estimated the percentage of the hypertensive population with each outcome—diagnosed, a missed opportunity for diagnosis (overall and, separately, at public and private facilities) and potentially diagnosed. We estimated these percentages by wealth group and covariates, with adjustment for age and sex. Adjustment used the age-sex distribution of the (representative) sample as the reference and was done by a) estimating a probit model for the respective outcome on indicators of sex and age group and, for example, wealth group, b) obtaining the predicted probability of each respondent having that outcome if they were in one of the wealth groups, and c) averaging these predictions across the (hypertensives) sample. Equal prevalence and equal diagnosis across categories of each covariate were tested using chi-squared tests and *P* values reported. We estimated fully adjusted differences in the probability of having each outcome by estimating a probit model for that outcome as a function of wealth group indicators, all the covariates and province/municipality indicators, and then averaging over the (hypertensives) sample the estimated difference in the probability between, for example, one wealth group and the reference group.

We also restricted the sample to those classified as hypertensive and not classified as diagnosed, and used a multinomial probit model to estimate fully adjusted differences by wealth group in the probabilities of a) not visiting any health facility (*no missed opportunity*), b) visiting a public facility (*missed opportunity at public*), and c) visiting a private facility (*missed opportunity at private*).

In supplementary analysis, we estimated, overall and by wealth group, the percentage of hypertensives in crisis (systolic BP ≥ 180 or diastolic BP ≥ 120) and percentages of those in crisis who were diagnosed and had a missed opportunity for diagnosis.

All analyses were done using *Stata* version 18.0. In all analyses, including the generation of wealth quintile groups, we applied sample weights to make the sample nationally representative of the older population after adjustment for systematic sampling of barangays, oversampling of the older old and non-response by age.^36^ We used survey methods in *Stata* (*svy*) to adjust 95% confidence intervals (CI) for sample stratification and clustering. The study was conducted and reported in accordance with Strengthening the Reporting of Observational Studies in Epidemiology (STROBE) guidelines (Supplementary Checklist S1).

### Ethical approval

LSAHP was approved by the University of the Philippines Manila Research Ethics Board Review Panel 2. Written informed consent was obtained from all respondents. No further ethical approval was required for the secondary analysis of LSAHP data conducted for this study.

### Role of the funding source

The funders had no role in the study design, its conduct, the interpretation and reporting of results, preparation of the manuscript and its submission for publication.

## Results

**Fig. 1:**
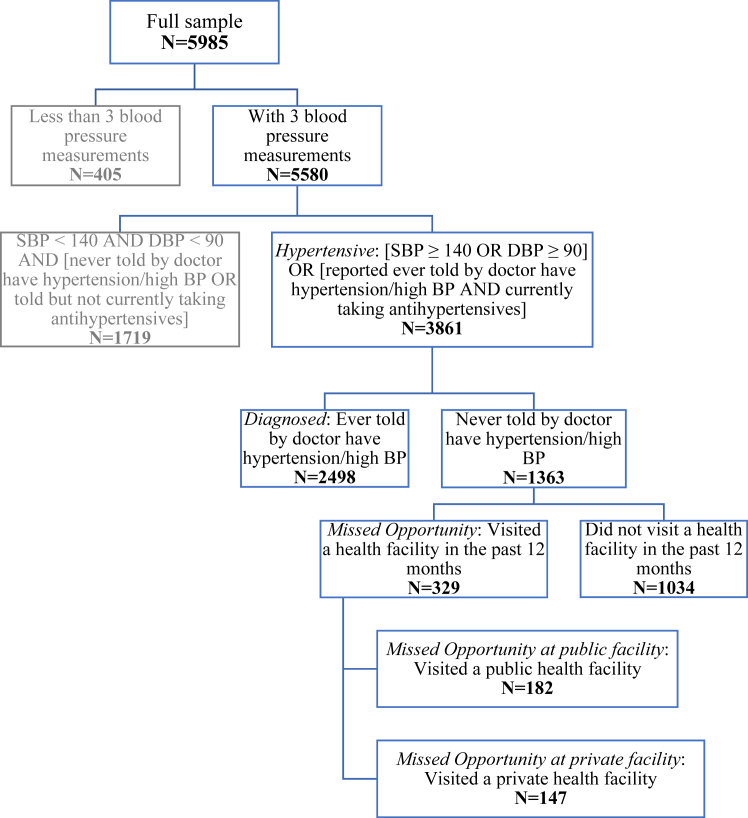
**Respondent Flow**.

Out of 5985 survey respondents, 5580 (93.2%) had their BP measured three times (Fig. 1). For most (366) of the 405 respondents dropped because of missing BP data there was no BP measurement at all. We estimated hypertension prevalence in the 60+ population of 67.1% (95% CI: 62.8, 71.3). Age- and sex-adjusted prevalence increased with wealth, although not entirely monotonically, and was also higher among females, urban residents and those not working (Supplementary Table S2).

Table 1 shows characteristics of the analysis sample of 3861 respondents classified as hypertensive: 62% were female, 35% were aged 60–64 years, 72% had no more than elementary education, 55% lived in rural areas, 57% lived with children, 57% were not working and 20% reported not having health insurance.

**Table 1:** Characteristics of the analysis sample of older (≥60 years) adults with hypertension.

	(n = 3861)	
	n	(%)
**Wealth quintile**		
Poorest	578	(16.1)
Poorer	845	(20.3)
Middle	802	(20.1)
Richer	844	(21.6)
Richest	792	(21.9)
**Education**		
Elementary or below	2565	(71.6)
High school/post-secondary	919	(20.6)
College and higher	377	(7.8)
**Age group, years**		
60–64	728	(35.3)
65–69	620	(26.6)
70–74	919	(14.9)
75–79	663	(11.4)
80+	931	(11.8)
**Sex**		
Female	2503	(62.2)
Male	1358	(37.8)
**Location**		
Rural	2130	(55.3)
Urban	1731	(44.7)
**Living arrangement**		
Living alone	518	(14.0)
Living w spouse only	371	(10.3)
Living w children	2312	(57.5)
Other types of arrangement	660	(18.1)
**Employment**		
Not working	2682	(57.1)
Working	1179	(42.9)
**Health insurance**		
No health insurance	879	(19.9)
Has health insurance	2982	(80.1)

*Note*. See Fig. 1 for sample selection.

Table 2 shows estimated percentages of those classified as hypertensive who a) were diagnosed, b) had a missed opportunity for diagnosis (at public/private facilities), and c) potentially would be diagnosed if missed opportunities were taken. We estimated that 62.7% (95% CI: 58.2, 67.0) of those classified as hypertensive had been diagnosed. The (age-sex adjusted) percentage diagnosed was lower in the bottom two wealth groups and among those with no more than elementary education. It was also lower among males and those living alone.

**Table 2:** Percentages diagnosed, with a missed opportunity for diagnosis and potentially diagnosed among older (≥60 years) adults with hypertension, Philippines 2018/19 (n = 3861).

	Diagnosed		Missed opportunity for diagnosis						Potentially diagnosed	
			Total		Public facility		Private facility			
	%	(95% CI)	%	(95% CI)	%	(95% CI)	%	(95% CI)	%	(95% CI)
**Overall**	62.7	(58.2, 67.0)	10.7	(7.7, 14.5)	6.7	(3.8, 11.0)	3.8	(2.6, 5.4)	74.4	(70.9, 77.6)
**Wealth***P*	*0.005*		*0.001*		*0.060*		*0.006*		*<0.001*	
Poorest	53.3	(46.2, 60.4)	3.89	(1.6, 7.8)	2.9	(1.0, 7.1)	0.9	(0.3, 2.0)	57.1	(49.7, 64.2)
Poorer	54.8	(45.8, 63.6)	10.9	(5.8, 18.5)	7.9	(3.5, 15.6)	2.8	(1.2, 5.9)	66.9	(58.3, 74.7)
Middle	62.4	(55.4, 68.9)	11.7	(6.1, 20.0)	7.1	(2.6, 16.0)	4.1	(1.8, 8.4)	75.3	(66.3, 82.8)
Richer	70.8	(63.3, 77.5)	9.0	(5.4, 14.1)	3.9	(1.7, 7.9)	4.8	(2.3, 9.1)	80.9	(72.9, 87.3)
Richest	69.1	(61.2, 76.2)	16.6	(9.9, 25.7)	10.7	(4.7, 20.9)	5.5	(3.1, 9.1)	87.0	(81.6, 91.2)
**Education***P*	*0.008*		*0.278*		*0.463*		*0.642*		*0.001*	
Elementary or less	58.5	(52.9, 63.9)	11.4	(8.0, 15.9)	7.2	(4.1, 12.0)	4.0	(2.4, 6.3)	71.1	(67.2, 74.8)
High school	72.9	(65.5, 79.4)	6.9	(4.3, 10.5)	4.0	(2.0, 7.3)	2.7	(1.3, 5.4)	80.5	(73.5, 86.2)
College	73.9	(60.8, 84.4)	14.1	(4.0, 34.8)	9.2	(1.1, 35.4)	4.7	(1.5, 11.8)	88.5	(80.4, 93.9)
**Age, years***P*	*0.072*		*<0.001*		*0.049*		*0.005*		*0.908*	
60–64	66.1	(58.9, 72.8)	6.7	(3.6, 11.3)	3.9	(1.5, 8.7)	2.7	(1.2, 5.8)	73.0	(67.2, 78.2)
65–69	55.7	(47.0, 64.0)	20.5	(12.9, 30.3)	12.4	(5.8, 23.3)	8.1	(4.8, 12.8)	76.3	(68.3, 83.1)
70–74	66.7	(59.1, 73.6)	7.2	(4.8, 10.3)	4.6	(2.6, 7.6)	2.5	(1.3, 4.4)	73.9	(66.1, 80.6)
75–79	60.4	(50.7, 69.5)	12.1	(6.1, 21.5)	10.1	(4.1, 20.9)	2.1	(0.8, 4.7)	72.6	(61.8, 81.7)
80+	64.5	(57.7, 70.9)	11.4	(6.9, 17.7)	7.1	(3.2, 14.0)	4.2	(2.7, 6.4)	76.4	(70.1, 81.9)
**Sex***P*	*0.058*		*0.698*		*0.953*		*0.613*		*0.059*	
Female	65.9	(60.5, 71.1)	10.4	(6.8, 15.2)	6.7	(3.3, 12.2)	3.6	(2.2, 5.4)	77.3	(72.2, 81.9)
Male	57.1	(49.4, 64.5)	11.2	(8.0, 15.4)	6.8	(4.3, 10.3)	4.1	(2.5, 6.6)	69.2	(63.2, 74.7)
**Location***P*	*0.504*		*0.002*		*0.001*		*0.7776*		*<0.001*	
Rural	61.2	(56.0, 66.3)	6.2	(4.3, 9.4)	2.5	(1.4, 4.3)	3.6	(2.0, 6.1)	68.1	(63.3, 72.6)
Urban	64.4	(56.4, 71.8)	16.4	(10.7, 24.3)	12.0	(6.4, 20.6)	4.0	(2.4, 6.4)	82.3	(79.2, 85.0)
**Living***P*	*0.206*		*0.570*		*0.089*		*0.861*		*0.107*	
Alone	50.2	(36.6, 63.8)	7.8	(3.5, 14.5)	3.1	(1.0, 8.1)	4.6	(1.7, 10.2)	59.2	(46.1, 71.3)
With spouse only	68.8	(57.3, 78.7)	7.4	(1.3, 14.7)	2.3	(0.8, 5.8)	4.9	(1.1, 15.4)	76.9	(68.1, 84.2)
With children	63.1	(56.8, 69.1)	12.7	(7.1, 20.2)	8.9	(4.2, 16.7)	3.6	(2.4, 5.2)	76.9	(72.1, 81.2)
Other	67.4	(59.0, 75.0)	8.3	(4.0, 14.5)	5.0	(2.2, 10.2)	3.0	(1.4, 6.1)	76.9	(69.1, 83.4)
**Employment***P*	*0.257*		*0.208*		*0.838*		*0.004*		*0.554*	
Not working	65.9	(58.3, 72.8)	8.7	(5.2, 13.8)	6.4	(3.0, 12.1)	2.2	(1.3, 3.4)	75.4	(71.0, 79.5)
Working	58.4	(49.8, 66.6)	13.5	(8.3, 20.6)	7.1	(2.9, 14.9)	6.1	(3.6, 9.8)	73.0	(66.3, 78.9)
**Health Insurance***P*	*0.920*		*0.035*		*0.095*		*0.267*		*0.211*	
No	63.0	(56.0, 69.6)	5.8	(3.4, 9.2)	3.0	(1.0, 6.4)	2.6	(1.4, 4.7)	70.0	(63.3, 76.0)
Yes	62.6	(57.4, 67.5)	11.9	(8.1, 16.9)	7.6	(4.0, 13.2)	4.0	(2.6, 6.1)	75.5	(70.9, 79.7)

*Note*. Sample restricted to those classified as *hypertensive* (Fig. 1). *Diagnosed* and *Missing Opportunity* (for diagnosis) as defined in Fig. 1. *Potentially diagnosed* = *Diagnosed* + *Missed Opportunity*. Percentages by covariates were adjusted for age and sex. *P* (values) for chi-squared of equal percentages across categories of the respective covariate.

We estimated that 19.7% (95% CI: 7.7, 14.5) of those classified as hypertensive were not diagnosed and yet had visited a health facility in the last 12 months. This prevalence of a missed opportunity for diagnosis was highest among the richest and lowest among the poorest. It was highest among those aged 65–69, reaching 20.5% (95% CI: 12.9, 30.3). Those living in urban locations and who reported having health insurance were most likely to have a missed opportunity for diagnosis. The *P*-value for the test of no difference in the probability of having a missed opportunity by education is greater than 0.1.

We estimated a higher prevalence of a missed opportunity for hypertension diagnosis at public facilities (6.7%; 95% CI: 3.8, 11.0) than at private facilities (3.8%; 95% CI: 2.6, 5.4), which reflected greater utilization of public facilities by OPs classified as having undiagnosed hypertension. Missed opportunities at public facilities occurred more often at hospitals than clinics, while their frequency was similar at private hospitals and clinics (Supplementary Table S3). Differences in missed opportunities by wealth and age were similar at public and private facilities. The higher likelihoods of urban dwellers and the insured to have a missed opportunity were observed only at public facilities. Those working were more likely to have a missed opportunity for diagnosis at private facilities.

We estimated that 74.4% (95% CI: 70.9, 77.6) of those classified as hypertensive could potentially have been diagnosed if opportunities had been taken to screen for hypertension at health facilities. Comparing percentages potentially diagnosed with percentages already diagnosed, opportunistic screening at health facilities would have increased inequalities in diagnosis by wealth, education, urban/rural location and health insurance cover, reduced inequalities by age and employment, and made little impact on inequalities by sex and living arrangement.

Table 3 shows fully adjusted percentage point (pp) differences in probabilities of being diagnosed, having a missed opportunity for diagnosis and being potentially diagnosed among those classified as hypertensive. We estimated that the poorest group were 11.0 pp (95% CI: −23.0, −0.9) less likely to be diagnosed than those in the richest group after adjusting for all covariates and province/municipality differences. Those with no more than elementary education were estimated to be 12.5 pp (95% CI: −23.9, −1.2) less likely to be diagnosed than the college educated. Males were 10.0 pp (95% CI: −19.1, −1.0) less likely to be diagnosed than females.

**Table 3:** Fully adjusted absolute differences in probabilities of being diagnosed, having a missed opportunity for diagnosis and being potentially diagnosed, older (≥60 years) adults with hypertension in the Philippines 2018/19 (n = 3861).

	Diagnosed		Missed opportunity for diagnosis		Potentially diagnosed	
	pp	(95% CI)	pp	(95% CI)	pp	(95% CI)
**Wealth**						
Poorest	−11.0	(−23.0, 0.9)	−7.6	(−14.7, −0.5)	−17.7	(−29.5, −5.9)
Poorer	−9.3	(−18.9, 0.2)	−1.1	(−9.0, 6.9)	−10.2	(−20.3, −0.2)
Middle	−0.8	(−9.9, 8.2)	−1.7	(−8.7, 5.2)	−4.8	(−14.8, 5.2)
Richer	5.6	(−2.5, 13.8)	−6.2	(−12.4, 0.0)	−2.8	(−11.5, 5.9)
Richest	Ref.		Ref.		Ref.	
**Education**						
Elementary or less	−12.5	(−23.9, −1.2)	2.3	(−6.1, 10.7)	−10.6	(−18.9, −2.3)
High school	−1.5	(−11.9, 9.0)	−3.9	(−12.9, 5.0)	−7.0	(−16.2, 2.2)
College	Ref.		Ref.		Ref.	
**Age, years**						
60–64	1.4	(−8.5, 11.2)	−10.8	(−18.4, −3.2)	−7.9	(−16.6, 0.8)
65–69	−6.2	(−17.1, 4.8)	2.0	(−7.4, 11.3)	−1.4	(−7.9, 5.1)
70–74	1.4	(−8.7, 11.5)	−7.4	(−15.7, 0.9)	−4.3	(−12.7, 4.1)
75–79	−5.4	(−15.8, 5.0)	−2.3	(−7.5, 3.0)	−6.4	(−16.0, 3.2)
80+	Ref.		Ref.		Ref.	
**Sex**						
Female	Ref.		Ref.		Ref.	
Male	−10.0	(−19.1, −1.0)	2.1	(−2.3, 6.6)	−8.8	(−16.2, −1.4)
**Location**						
Rural	Ref.		Ref.		Ref.	
Urban	−6.2	(−14.6, 2.1)	6.5	(−1.3, 14.3)	0.9	(−6.2, 7.9)
**Living arrangement**						
Alone	−12.8	(−31.9, 6.2)	0.7	(−5.2, 6.5)	−10.4	(−26.5, 5.7)
With spouse only	1.0	(−11.3, 13.3)	1.3	(−7.7, 10.3)	3.5	(−5.5, 12.6)
With children	−6.8	(−19.2, 5.7)	4.6	(−4.0, 13.2)	−0.7	(−6.8, 5.5)
Other	Ref.		Ref.		Ref.	
**Employment**						
Not working	Ref.		Ref.		Ref.	
Working	−4.8	(−16.1, 6.4)	7.4	(1.2, 13.5)	1.7	(−5.3, 8.8)
**Health Insurance**						
No	Ref.		Ref.		Ref.	
Yes	−1.0	(−9.5, 7.5)	3.7	(−2.4, 9.7)	1.5	(−7.3, 10.3)

*Note*. Sample restricted to those classified as *hypertensive* (Fig. 1). *Diagnosed* and *Missing Opportunity for diagnosis* as defined in Fig. 1. *Potentially diagnosed* = *Diagnosed* + *Missed Opportunity*. Estimates are averaged, adjusted, absolute risk differences between each group and the respective reference group obtained from a probit model of each outcome as a function of all covariates, plus city/province indicators. pp, percentage point. Ref., reference group.

The poorest fifth were 7.6 pp (95% CI: −14.7, −0.5) less likely than the richest fifth to have a missed opportunity for diagnosis. The second richest group had the second lowest probability of a missed opportunity. After full adjustment, there were no differences in the likelihood of having a missed opportunity across education groups. The youngest group of OPs (aged 60–64) were estimated to be 10.8 pp (95% CI: −18.4, −3.2) less likely to have a missed opportunity than the oldest group (80+), and the point estimates continue to show the highest likelihood of a missed opportunity among those aged 65–69. Workers were estimated to be 7.4 pp (95% CI: 1.2, 13.5) more likely than non-workers to have a missed opportunity.

Comparing fully adjusted differences in the probabilities of being diagnosed and potentially diagnosed reveals that opportunistic screening would increase inequality in hypertension diagnosis by wealth. For example, we estimated that if those with a missed opportunity had been diagnosed, then the poorest would have been 17.7 pp (95% CI: −29.5, −5.9) less likely than the richest to be diagnosed, an increase in magnitude of almost 7 pp compared with the respective estimated difference in the actual diagnosis rate. The estimates suggest that opportunistic screening would only slightly reduce differences in diagnosis by education and sex, which would remain to the disadvantage of the less educated and males.

Table 4 shows, for OPs classified as hypertensive and undiagnosed, fully adjusted differences by wealth group in the probabilities of visiting no health facility, a public facility or a private facility (Supplementary Table S4 for full model estimates). Within this sample, those who visited a facility missed an opportunity for diagnosis. We estimated that the poorest undiagnosed OPs were 24.9 pp (95% CI: 9.0, 40.8) more likely than the richest undiagnosed OPs not to visit a health facility. This difference is mostly explained by the poorest being 19.1 pp (95% CI: −30.1, −8.1) less likely to have visited a private facility. Poorer OPs were less likely to have had a missed opportunity for diagnosis because they were less likely to go for private healthcare.

**Table 4:** Fully adjusted absolute differences in probabilities of visiting health facilities by wealth group, undiagnosed older (≥60 years) adults with hypertension in the Philippines 2018/19 (n = 1363).

	Health facility visited in last 12 months					
	None		Public		Private	
	pp	(95% CI)	pp	(95% CI)	pp	(95% CI)
**Wealth**						
Poorest	24.9	(9.0, 40.8)	−5.8	(−20.8, 9.2)	−19.1	(−30.1, −8.1)
Poorer	12.8	(−2.9, 28.5)	0.9	(−11.2, 13.0)	−13.7	(−26.1, −1.3)
Middle	8.7	(−6.2, 23.6)	0.9	(−11.9, 13.7)	−9.6	(−20.6, 1.4)
Richer	7.6	(−6.6, 21.8)	−8.5	(−20.5, 3.4)	0.9	(−11.1, 13.0)
Richest	Ref.		Ref.		Ref.	

*Note*. Sample restricted to those classified as *hypertensive* and not classified as *diagnosed,* both as defined in Fig. 1. Estimates are averaged, adjusted, absolute risk differences between each group and the reference group obtained from a multinomial probit model of health facility visited (none, public or private) as a function of the wealth group indicators, all the covariates in Table 3, plus city/province indicators. Supplementary Table S4 gives estimates for all covariates in the model. pp, percentage point difference. Ref., reference group.

We estimated that 8.4% (95% CI: 6.7, 10.4) of hypertensives were in BP crisis, with higher percentages among poorer groups in the sample (Supplementary Table S5). Among those in crisis, the percentage diagnosed (67.3%, 95% CI: 57.8, 75.9) was higher and the percentage with a missed opportunity (1.1%, 95% CI: 0.6, 2.0) was much lower than among all hypertensives (Supplementary Table S5). Among those in crisis, the richest were also most likely to have been diagnosed but they were not most likely to have had a missed opportunity for diagnosis.

## Discussion

Consistent with other studies,^37^, ^38^, ^39^ we found high (around two thirds) prevalence of hypertension in the older (60+) population of the Philippines, which was even higher in wealthier groups, and low diagnosis of the condition, which was even lower in poorer groups. These patterns are consistent with evidence from India,^17^ although the direction of socioeconomic inequality in hypertension prevalence varies across middle-income countries.^40^^,^^41^

Almost two fifths of those classified as hypertensive remained undiagnosed. The combination of high prevalence and low diagnosis implies that many older Filipinos are unwittingly exposed to substantial risk from CVDs, including from heart attack and stroke.^2^^,^^42^, ^43^, ^44^, ^45^ Increasing early diagnosis of hypertension is a critical first step to reducing these risks. Given the general high utilization of healthcare by the elderly, opportunistic screening for hypertension at health facilities can potentially raise diagnosis rates and improve primary prevention of CVDs.

We found that this screening could increase the rate of diagnosis of the older, hypertensive population of the Philippines by almost twelve percentage points. While this is lower, both absolutely and relatively, than the estimated impact of opportunistic screening on hypertension diagnosis in the older (45+) population of India,^34^ it could still translate into substantial reductions in CVD related morbidity and mortality, provided it were followed by effective management of diagnosed hypertension to bring blood pressure under control.

### Health system and policy implications

Given a population of 8.7 million older persons in the Philippines in 2018,^46^ our estimates imply that opportunistic screening would increase the number with diagnosed hypertension from 3.6 million to 4.3 million. It would generate an additional 676,000 diagnosed hypertension patients aged 60+ that would need to be managed through maintenance medication, lifestyle counselling and regular check-up consultations. This would be a substantial challenge for a tightly constrained health system. Maintenance medications for hypertensives are currently provided without charge at public health clinics through Hypertension and Diabetes Clubs.^26^ They are included in the public health insurance primary care benefit package, which, in principle, also covers initial and follow-up primary care consultations, as well as health screening and assessment, including selected diagnostic services.^47^ Antihypertensives are also part of a proposed public insurance outpatient medicine benefit package.^48^ Management of the additional patients would require faster roll out and scale up of this benefit package and increased budgetary provision for the procurement of medicines. Our estimates suggest that approximately 431,000 of the additional patients would be diagnosed at public facilities, while another 245,000 would be diagnosed at private facilities. Some proportion of the latter number may be expected to resort to the public health system for hypertension management given the statutory right to free maintenance medicines at public clinics. Cost-effectiveness of opportunistic screening for cardiovascular disease risk (including hypertension) has been demonstrated in another middle-income country (Sri Lanka).^49^ Modelling the cost of opportunistic hypertension screening and setting it against resources saved through prevented disease would be valuable to health system decision makers in the Philippines and other Western Pacific countries.

Beyond consideration of the health system investment necessary to meet the needs of substantially more hypertension patients generated by opportunistic screening, our findings also raise the question of why so many older hypertensive Filipinos remain undiagnosed despite recent contact with health facilities. According to the WHO PEN protocol (PhilPEN in the Philippines),^25^ all older persons visiting a public health clinic should have been screened for CVD risk and put on antihypertensives if their risk or BP was high. Our results suggest that there are substantial deficiencies in the operation of this opportunistic screening, which is consistent with evidence of poor implementation of hypertension screening guidelines elsewhere in the Western Pacific.^50^ Possible reasons for this gap between policy and practice, which we cannot test with the data available, include understaffing of public clinics that are hard-pressed even to respond to the complaints with which patients present without also screening for hypertension that may not be an immediate concern. In principle, there should be sufficient supply of screening equipment and of medicines to managed cases detected. All public clinics were equipped with blood pressure monitors as part of the PhilPEN implementation^25^ and the clinics should receive a supply of maintenance medicines to manage hypertension.^26^ However, drug stockouts are not uncommon. A randomized experiment on a study population aged 40–70 with no history of CVD in one province of the Philippines found that visiting a public clinic responsible for operating PhilPEN increased the probability of having blood pressure measured, but it did not increase the probabilities of being diagnosed with hypertension or being put on antihypertensives.^27^ This serves as a warning that we estimated the increase in hypertension diagnosis potentially achievable by effectively implemented opportunistic screening. Besides ensuring that clinics have sufficient capacity to respond to increased demand for hypertension management and medication, it may be necessary to ensure that their incentives to deliver that care are aligned with the interests of patients living with undiagnosed hypertension.

### Implications for inequality

Our estimates imply that opportunistic screening, if implemented effectively, would increase diagnosis of hypertension across all wealth groups. The increase in diagnosis would, however, be smallest—absolutely and relatively—among the poorest. Effective opportunistic screening at health facilities would exacerbate inequality in the diagnosis of hypertension. The rate of undiagnosed hypertension would remain above 40% among the poorest fifth of the older population. This negative impact on inequality in diagnosis reflects inequality in the utilization of healthcare by older Filipinos with undiagnosed hypertension. The economically worst off in this subpopulation are least likely to visit a health facility and so would have least opportunity to be diagnosed even if facilities were systematically screening all older patients for hypertension.

Inequality in utilization is mainly driven by the poor being less likely to visit private health facilities, without them having any offsetting increased likelihood of visiting public facilities. To raise the rate of diagnosis in this hard-to-reach group of poor hypertensives, it may be necessary to lower barriers impeding access to both public and private care. Of an estimated 29% of older Filipinos who did not visit a health facility despite being sick, a majority cited a lack of financial means as the reason.^51^ Half of the population cannot access a public clinic within 30 min.^52^ Less than five percent of public health spending on NCDs goes to primary healthcare and only about one percent of the expenditure of the public health insurance corporation is on its primary care benefit package.^52^ With such low investment in primary care, it is not surprising that many people with chronic health problems go directly to public and private hospitals.^52^ But given the higher travel and direct costs of using hospital care, it is even less accessible to the poorest older people who live in rural locations.

One way to increase the diagnosis of hypertension in this group, and to improve management of the condition, would be to expand the public insurance primary care benefit package to cover treatment received at private clinics. Increasing awareness of health insurance coverage may also help. In principle, all of the population aged 60+ has public health insurance. Yet, we found that around one fifth of those with hypertension reported not having health insurance and they were less likely to have visited a health facility and so to have a missed opportunity for diagnosis. There is a risk that the poorest elderly would continue to live with undiagnosed hypertension even if opportunistic screening were effectively implemented at health facilities because they are not aware that they have insurance to cover costs of a healthcare visit and any maintenance medication prescribed as a result.

Another strategy to avoid opportunistic screening exacerbating inequality in hypertension diagnosis would be to conduct it in the community, e.g. at pharmacies or retailers frequented by poorer OPs, as well as at health facilities. While this would increase cost, it is likely to be more cost-effective than population-based screening.

### Strengths and limitations

The strength of this study is the use of data that are nationally representative of the older population, with a sample size that was sufficient to stratify by economic status and examine inequality in diagnosis and missed opportunities for diagnosis. It adds to the many studies undertaken of hypertension awareness (diagnosis), treatment and control by also using data on healthcare utilization to estimate the potential effect on diagnosis of opportunistic screening.

The study's main limitation is that blood pressure was measured when respondents were asked about visits to health facilities in the past 12 months. Some of those who had high blood pressure at the time of the survey interview would not necessarily have had high blood pressure when they used healthcare in the previous year. For that reason, like other studies,^32^^,^^33^^,^^49^ we may have overestimated the increase in hypertension diagnosis that could be achieved by opportunistic screening. On the other hand, some of those who did not have high blood pressure at the time of the survey, would have had high blood pressure when earlier using healthcare. To an extent, one bias will offset the other. Blood pressure that is temporarily high at the time of the survey may partly explain why wealthier individuals are identified as having missed opportunities for diagnosis. Additionally, this can be because these patients also do not have their blood pressure routinely taken when they present with an apparently unrelated condition.

Following standard practice in the estimation of hypertension prevalence,^6^^,^^7^ we classified a respondent as hypertensive if they had high blood pressure at the time of the survey or they were taking medication for high blood pressure at that time. Clinical diagnosis of hypertension is often done on the basis of blood pressure readings taken on more than one occasion.^32^ This discrepancy may have caused this study, like others based on survey data, to overestimate hypertension prevalence. Again, there will have been an offsetting effect from hypertensive respondents who were measured with blood pressure below the hypertension thresholds at the time of the survey.

The analysis sample excluded 405 respondents whose blood pressure could not be measured, most often because consent was not given but also, in some cases, because a monitor could not be fitted. The latter occurred with some obese respondents because monitors with extra-large cuffs were not initially available to enumerators. This will have biased the estimate of hypertension prevalence slightly downward. Implications for estimates of diagnosis and missed opportunities are not obvious. Another limitation was the relatively long (12-month) recall period for a health facility visit that may have increased the likelihood that respondents forgot visits. This would have pushed bias in the direction of underestimation of missed opportunities for diagnosis, also offsetting any overestimation from false positives in those classified as hypertensive. A final data limitation is that while the sample size was sufficient to estimate diagnosis and missed opportunities for diagnosis among hypertensives overall and by equal-sized wealth groups, it did constrain the precision of estimates of missed opportunities at public and private facilities and in smaller categories of some covariates.

A deliberate limitation is that the study confined attention to diagnosis, ignoring deficiencies in treatment and control of hypertension, which were examined in another paper based on the same data.^37^

### Conclusion

This study demonstrated that opportunistically screening older Filipinos for hypertension at health facilities has strong potential to increase diagnosis of a condition that, as the principal risk factor for CVDs, poses a major risk to health and survival at older ages unless it is detected early and brought under control through highly cost-effective medicines. Such screening could ensure that around three quarters of older people living with hypertension are diagnosed. Poorer people account for a disproportionate share of those who would remain undiagnosed. In fact, our estimates suggest that, due to inequalities in healthcare utilization, opportunistic screening at health facilities would exacerbate inequality in the diagnosis of hypertension. Avoiding this scenario would likely require policy interventions to lower geographic, economic and information barriers that poor older Filipinos face in accessing primary care delivered by both public and private facilities. It may also require complementing opportunistic screening for hypertension at health facilities with community-based screening targeted at hard-to-reach poor and rural older populations.

## Contributors

GTC conceptualized the survey, supervised the data collection, provided inputs to the initial draft and reviewed final draft of the paper. OO’D conceptualized the study. GTC, KGRC and ADK had access to and verified the raw data. KGRC processed the data and performed the statistical analysis. KGRC prepared the tables, figures and supplementary materials. ADK, JJC and OO’D analyzed and interpreted the data, drafted and finalized the manuscript. OO’D had final responsibility to submit for publication.

## Data sharing statement

The data from this study are available upon reasonable request here https://www.drdf.org.ph/lsahp-baseline-data-request-portal/.

## Declaration of interests

The authors declare that they have no conflicts of interest. That is, they have no relationship, activity or interest listed on the ICMJE COI form (signed by each author) that is related to the content of this manuscript, where “related” means any relation with for-profit or not-for-profit third parties whose interests may be affected by the content of the manuscript.
