# Supplementary material for: Missed opportunities for hypertension screening of older people in the Philippines: cross-sectional analysis of nationally representative individual-level data
Source: Lancet Reg Health West Pac. 2024 Sep 4;50:101188. doi: 10.1016/j.lanwpc.2024.101188 (PMC11407953; doi:10.1016/j.lanwpc.2024.101188)
Supplement: Supplementary Tables [file mmc1.docx]

**Missed opportunities for hypertension screening of older people in the Philippines: cross-sectional analysis of nationally representative individual-level data**

Aleli D. Kraft, Joseph J. Capuno, Kayleen Gene R. Calicdan

Grace T. Cruz, Owen O’Donnell

**Correspondence:** Owen O'Donnell, Erasmus School of Health Policy and Management, PO Box 1738, 3000 DR Rotterdam, The Netherlands. [odonnell@ese.eur.nl](mailto:odonnell@ese.eur.nl) T: +30 2313 007340

**Supplementary Material**

**Table of Contents**

| **Table** | **Content** | **Page** |
| --- | --- | --- |
| S1 | Means and standard deviation of indicators included in the principal components analysis (PCA) and their PCA weights used to construct the wealth index | 2 |
| S2 | Hypertension prevalence in the older (60+) population of the Philippines, 2018/19 | 3 |
| S3 | Percentages with a missed opportunity for diagnosis at public/private and clinic/hospital facilities among older (≥ 60 years) adults with hypertension) | 4 |
| S4 | Fully adjusted absolute differences in probabilities of visiting health facilities by wealth group, undiagnosed older (≥ 60 years) adults with hypertension in the Philippines 2018/19 (n=1363) | 5 |
| S5 | Hypertensive crisis (SBP ≥ 180 or DBP ≥ 120) among those with hypertension and diagnosis and missed opportunity for diagnosis among those in crisis, older (≥ 60 years) adults, Philippines 2018/19 | 6 |
| Checklist S1 | STROBE Statement for cross-sectional study | 7-10 |
| References |  | 10 |

| **Table S1**. Means and standard deviation of indicators included in the principal components analysis (PCA) and their PCA weights used to construct the wealth index (n=5580) | | | | |
| --- | --- | --- | --- | --- |
|  | Mean | SD | Weight | Weight/  SD |
| Household owns (yes = 1, no = 0) |  |  |  |  |
| Car | 0.044 | 0.206 | 0.042 | 0.202 |
| Motorcycle/tricycle | 0.239 | 0.427 | 0.053 | 0.124 |
| Motorized boat/banca | 0.032 | 0.176 | 0.002 | 0.011 |
| Air conditioner | 0.082 | 0.275 | 0.059 | 0.213 |
| Washing machine | 0.336 | 0.472 | 0.083 | 0.177 |
| Stove | 0.174 | 0.379 | 0.057 | 0.150 |
| Refrigerator | 0.388 | 0.487 | 0.089 | 0.183 |
| Computer | 0.105 | 0.306 | 0.060 | 0.196 |
| Cellular/mobile phone | 0.660 | 0.474 | 0.072 | 0.152 |
| Landline/wireless phone | 0.038 | 0.192 | 0.041 | 0.216 |
| Audio component/stereo set | 0.127 | 0.333 | 0.048 | 0.144 |
| Karaoke/videoke/Magic Sing | 0.062 | 0.242 | 0.035 | 0.144 |
| CD/VCD/DVD player | 0.203 | 0.402 | 0.053 | 0.131 |
| Television | 0.717 | 0.450 | 0.086 | 0.190 |
| Radio/cassette player | 0.351 | 0.477 | 0.021 | 0.045 |
| Household has electricity | 0.922 | 0.268 | 0.059 | 0.222 |
| Household has internet | 0.164 | 0.370 | 0.066 | 0.179 |
| House/lot ownership/tenure |  |  |  |  |
| Own house and lot (or owner-like possession) | 0.660 | 0.474 | 0.042 | 0.089 |
| Own house, rent lot | 0.031 | 0.172 | 0.000 | 0.001 |
| Own house, rent-free lot with consent of owner | 0.190 | 0.393 | -0.049 | -0.125 |
| Own house, rent-free lot without consent of owner | 0.019 | 0.137 | -0.006 | -0.040 |
| Household source of drinking water |  |  |  |  |
| Piped into dwelling | 0.230 | 0.421 | -0.031 | -0.074 |
| Piped to yard/plot | 0.033 | 0.180 | -0.040 | -0.225 |
| Piped to neighbor | 0.038 | 0.190 | -0.059 | -0.309 |
| Piped to public tap/stand pipe | 0.087 | 0.282 | -0.075 | -0.264 |
| Tubed well/borehole | 0.094 | 0.292 | -0.060 | -0.207 |
| Protected dug well/Protected spring | 0.087 | 0.282 | -0.077 | -0.272 |
| Unrotected dug well/Unprotected spring/Rainwater/Tankertruck/Cart with small tank/Surface water | 0.018 | 0.134 | -0.031 | -0.232 |
| Refilling station/Bottled water | 0.413 | 0.492 | 0.000 | 0.000 |
| Household source of water for other uses |  |  |  |  |
| Piped into dwelling | 0.544 | 0.498 | 0.000 | 0.000 |
| Piped to yard/plot | 0.052 | 0.223 | -0.056 | -0.253 |
| Piped to neighbor | 0.041 | 0.198 | -0.064 | -0.325 |
| Public taps/stand pipe | 0.092 | 0.288 | -0.084 | -0.291 |
| Tubed well/borehole | 0.128 | 0.334 | -0.078 | -0.233 |
| Protected dug well/Protected spring | 0.098 | 0.297 | -0.087 | -0.294 |
| Unrotected dug well/Unprotected spring/Rainwater/Tankertruck/Cart with small tank/Surface water | 0.046 | 0.208 | -0.061 | -0.295 |
| Household sanitation |  |  |  |  |
| Flush to piped sewer system | 0.008 | 0.088 | -0.013 | -0.147 |
| Flush to septic tank | 0.890 | 0.313 | 0.000 | 0.000 |
| Flush to pit latrine | 0.036 | 0.186 | -0.069 | -0.369 |
| Flush to somewhere else/Flush to don't know where | 0.004 | 0.067 | -0.021 | -0.320 |
| Non-flush/other types of toilet/no toilet | 0.062 | 0.242 | -0.112 | -0.463 |
| Roof |  |  |  |  |
| Made of strong or mixed but predominantly strong materials | 0.875 | 0.331 | 0.075 | 0.226 |
| Made of light or mixed but predominantly light materials | 0.116 | 0.320 | -0.072 | -0.226 |
| Made of salvaged or mixed but predominantly salvaged materials | 0.010 | 0.098 | -0.017 | -0.175 |
| Outer walls |  |  |  |  |
| Made of strong or mixed but predominantly strong materials | 0.700 | 0.458 | 0.096 | 0.210 |
| Made of light or mixed but predominantly light materials | 0.271 | 0.444 | -0.089 | -0.201 |
| Made of salvaged or mixed but predominantly salvaged materials | 0.027 | 0.163 | -0.027 | -0.166 |
| Floor |  |  |  |  |
| Natural flooring | 0.053 | 0.225 | -0.077 | -0.345 |
| Rudimentary flooring | 0.183 | 0.387 | -0.138 | -0.358 |
| Finished flooring - Ceramic tiles | 0.197 | 0.398 | 0.000 | 0.000 |
| Finished flooring - Cement | 0.544 | 0.498 | -0.064 | -0.128 |
| Finished flooring - Others | 0.023 | 0.151 | -0.024 | -0.162 |
| Natural flooring | 0.053 | 0.225 | -0.077 | -0.345 |
| 4P beneficiary | 0.130 | 0.337 | 0.000 | 0.000 |
| **Wealth Index** | <0.001 | 1.00 |  |  |

| **Table S2.** Hypertension prevalence in the older (60+) population of the Philippines, 2018/19 | | | | |
| --- | --- | --- | --- | --- |
|  | (n=5,580) | | | |
|  | % | (95% CI) | | *P* |
| **Overall** | 67.1 | (62.8, | 71.3) |  |
| **Wealth quintile** |  |  |  | *0.006* |
| Poorest | 53.4 | (44.1, | 62.6) |  |
| Poorer | 69.2 | (63.5, | 74.5) |  |
| Middle | 65.6 | (57.8, | 72.8) |  |
| Richer | 73.6 | (66.0, | 80.3) |  |
| Richest | 74.1 | (67.1, | 80.2) |  |
| **Education** |  |  |  | *0.421* |
| Elementary or below | 65.6 | (60.3, | 70.6) |  |
| High school/post-secondary | 72.6 | (67.5, | 77.2) |  |
| College and higher | 67.9 | (50.3, | 82.2) |  |
| **Age group, years** |  |  |  | *0.468* |
| 60-64 | 66.3 | (57.9, | 73.9) |  |
| 65-69 | 64.9 | (56.8, | 72.4) |  |
| 70-74 | 67.2 | (60.3, | 73.5) |  |
| 75-79 | 69.8 | (64.0, | 75.1) |  |
| 80+ | 72.6 | (67.1, | 77.5) |  |
| **Sex** |  |  |  | *0.008* |
| Female | 69.8 | (66.0, | 73.4) |  |
| Male | 63.0 | (56.3, | 69.4) |  |
| **Location** |  |  |  | *0.045* |
| Rural | 63.9 | (57.7, | 69.7) |  |
| Urban | 71.7 | (66.8, | 76.2) |  |
| **Living Arrangement** |  |  |  | *0.072* |
| Living alone | 68.7 | (61.0, | 75.7) |  |
| Living w spouse only | 75.7 | (67.3, | 82.8) |  |
| Living w children | 64.0 | (57.6, | 70.0) |  |
| Other types of arrangement | 72.6 | (64.6, | 79.6) |  |
| **Employment** |  |  |  | *0.011* |
| Not working | 71.3 | (67.0, | 75.2) |  |
| Working | 62.4 | (55.2, | 69.1) |  |
| **Health Insurance** |  |  |  | *0.744* |
| No health insurance | 68.4 | (59.1, | 76.7) |  |
| Has health insurance | 66.8 | (62.3, | 71.1) |  |
| *Note.* Hypertension defined in Figure 1. Prevalence estimated from the sample of 5,580 respondents with three blood pressure measurements. Prevalence by covariates adjusted for age and sex. *P* (values) for chi-squared test of equal prevalence across categories of the respective covariate | | | | |

| **Table S3.** Percentages with a missed opportunity for diagnosis at public/private and clinic/hospital facilities  among older (≥ 60 years) adults with hypertension, Philippines 2018/19 (n=3861) | | | | | | | | |
| --- | --- | --- | --- | --- | --- | --- | --- | --- |
|  | Public | | | | Private | | | |
|  | Clinic | | Hospital | | Clinic | | Hospital | |
|  | pp | (95% CI) | pp | (95% CI) | pp | (95% CI) | pp | (95% CI) |
| Overall | 1.5 | (0.9, 2.4) | 4.0 | (1.7, 8.3) | 1.8 | (1.0, 3.0) | 1.6 | (1.0, 2.5) |
|  |  | |  | |  | |  | |
| Wealth *P* | *0.001* | | *0.686* | | *0.007* | | *0.103* | |
| Poorest | 0.4 | (0.0, 1.9) | 2.3 | (0.6, 7.0) | 0.2 | (0.0, 0.7) | 0.6 | (0.2, 1.6) |
| Poorer | 2.1 | (0.6, 6.0) | 4.7 | (1.1, 14.5) | 0.6 | (0.2, 1.6) | 2.0 | (0.7, 5.2) |
| Middle | 1.1 | (0.4, 2.6) | 4.8 | (2.3, 9.1) | 2.8 | (1.0, 7.0) | 0.9 | (0.4, 2.0) |
| Richer | 0.2 | (0.0, 1.2) | 3.1 | (1.1, 7.3) | 3.0 | (1.2, 6.7) | 1.3 | (0.6, 2.7) |
| Richest | 3.7 | (1.3, 8.7) | 4.7 | (1.2, 13.6) | 2.0 | (0.9, 4.3) | 3.0 | (1.3, 6.3) |
| Education *P* | *0.008* | | *0.310* | | *0.051* | | *0.117* | |
| Elementary or less | 1.7 | (0.9, 3.0) | 4.1 | (2.0, 7.6) | 2.0 | (1.0, 4.0) | 1.5 | (0.9, 2.5) |
| High school | 1.4 | (0.4, 4.1) | 1.8 | (0.8, 3.9) | 1.6 | (0.6, 3.9) | 0.8 | (0.3, 2.2) |
| College | 0.1 | (0.0, 0.5) | 8.7 | (1.0, 34.3) | 0.4 | (0.1, 1.3) | 4.4 | (1.2, 12.3) |
| Age, years *P* | *0.015* | | *0.032* | | *0.002* | | *0.056* | |
| 60-64 | 0.3 | (0.1, 0.9) | 3.4 | (1.0, 9.2) | 2.0 | (0.6, 5.2) | 0.7 | (0.3, 1.8) |
| 65-69 | 6.3 | (1.8, 16.5) | 5.6 | (1.9, 13.4) | 4.4 | (2.2, 8.2) | 3.4 | (1.5, 7.1) |
| 70-74 | 2.2 | (0.8, 5.2) | 2.0 | (1.1, 3.5) | 0.6 | (0.2, 1.7) | 1.8 | (0.8, 3.8) |
| 75-79 | 0.6 | (0.2, 1.7) | 9.0 | (3.4, 19.9) | 0.5 | (0.2, 1.2) | 1.5 | (0.4, 4.1) |
| 80+ | 3.5 | (1.3, 8.4) | 2.8 | (1.0, 7.0) | 1.5 | (0.8, 2.9) | 2.6 | (1.4, 4.4) |
| Sex *P* | *0.132* | | *0.274* | | *0.113* | | *0.370* | |
| Female | 2.4 | (1.0, 5.1) | 3.2 | (0.9, 8.9) | 1.4 | (0.7, 2.7) | 1.9 | (1.1, 3.0) |
| Male | 0.6 | (0.2, 2.0) | 5.6 | (3.3, 9.0) | 2.6 | (1.4, 4.6) | 1.3 | (0.5, 2.7) |
| Location *P* | *0.087* | | *0.004* | | *0.402* | | *0.063* | |
| Rural | 0.6 | (0.2, 2.1) | 1.4 | (0.7, 2.6) | 2.2 | (0.9, 4.5) | 1.1 | (0.5, 2.0) |
| Urban | 2.8 | (1.3, 5.7) | 7.3 | (2.7, 16.2) | 1.3 | (0.6, 2.6) | 2.3 | (1.3, 4.1) |
| Living Arrangement *P* | *0.382* | | *<0.001* | | *0.647* | | *0.510* | |
| Alone | 1.0 | (0.1, 5.3) | 1.5 | (0.3, 5.6) | 0.9 | (0.2, 3.2) | 3.3 | (1.0, 8.7) |
| With spouse only | 0.3 | (0.0, 2.3) | 1.6 | (0.4, 5.1) | 3.3 | (0.4, 14.3) | 1.2 | (0.4, 3.6) |
| With children | 1.6 | (0.9, 2.9) | 5.9 | (2.3, 13.0) | 2.0 | (1.2, 3.1) | 1.3 | (0.7, 2.3) |
| Other | 2.4 | (1.0, 4.9) | 1.4 | (0.3, 4.7) | 1.0 | (0.2, 3.8) | 1.7 | (0.8, 3.3) |
| Employment *P* | *0.779* | | *0.414* | | *0.073* | | *0.006* | |
| Not working | 1.7 | (0.6, 4.0) | 3.2 | (2.0, 5.0) | 1.1 | (0.6, 1.9) | 0.9 | (0.4, 1.6) |
| Working | 1.3 | (0.4, 3.7) | 5.0 | (1.3, 14.5) | 2.8 | (1.2, 6.0) | 2.8 | (1.6, 4.7) |
| Health Insurance *P* | *0.001* | | *0.458* | | *0.525* | | *0.307* | |
| No | 0.1 | (0.0, 0.5) | 2.7 | (1.0, 6.2) | 1.3 | (0.5, 3.0) | 1.1 | (0.4, 2.4) |
| Yes | 1.9 | (1.1, 3.1) | 4.3 | (1.7, 9.7) | 1.9 | (1.0, 3.6) | 1.8 | (1.1, 2.8) |
| *Note*. Table shows percentages with a missed opportunity for hypertension diagnosis at four types of health facilities. Public clinics include barangay health stations and rural health units. Public hospitals include municipal/community, district, provincial/city, regional, national and public specialty hospitals. Sample is restricted to those classified as *hypertensive* as defined in **Figure 1**. Missing opportunity for diagnosis as defined in Figure 1. Percentages by covariates are adjusted for age and sex. *P* (values) for chi-squared test of equal proportions across categories of the respective covariate. | | | | | | | | |

| **Table S4.** Fully adjusted absolute differences in probabilities of visiting health facilities by wealth group, undiagnosed older (≥ 60 years) adults with hypertension in the Philippines 2018/19 (n=1363) | | | | | | | | | |
| --- | --- | --- | --- | --- | --- | --- | --- | --- | --- |
|  | **Undiagnosed, did not**  **visit any health facility** | | | **Missed Opportunity (Public)** | | | **Missed Opportunity (Private)** | | |
|  | pp | 95% CI | *P* | pp | 95% CI | *P* | pp | 95% CI | *P* |
| **Wealth quintile** |  |  |  |  |  |  |  |  |  |
| Poorest | 24.9 | (9.0, 40.8) | *0.002* | -5.8 | (-20.8, 9.2) | *0.444* | -19.1 | (-30.1, -8.1) | *0.001* |
| Poorer | 12.8 | (-2.9, 28.5) | *0.110* | 0.9 | (-11.2, 13.0) | *0.879* | -13.7 | (-26.1, -1.3) | *0.030* |
| Middle | 8.7 | (-6.2, 23.6) | *0.252* | 0.9 | (-11.9, 13.7) | *0.893* | -9.6 | (-20.6, 1.4) | *0.087* |
| Richer | 7.6 | (-6.6, 21.8) | *0.291* | -8.5 | (-20.5, 3.4) | *0.160* | 0.9 | (-11.1, 13.0) | *0.880* |
| Richest | Ref. | | | Ref. | | | Ref. | | |
| **Education** |  |  |  |  |  |  |  |  |  |
| Elementary or below | 7.8 | (-12.5, 28.1) | *0.451* | -7.2 | (-27.2, 12.8) | *0.477* | -0.5 | (-10.9, 9.8) | *0.917* |
| High school/Vocational | 15.2 | (-9.0, 39.4) | *0.217* | -11.6 | (-36.6, 13.5) | *0.363* | -3.6 | (-14.5, 7.3) | *0.514* |
| College and higher | Ref. | | | Ref. | | | Ref. | | |
| **Age group** |  |  |  |  |  |  |  |  |  |
| 60-64 | 24.2 | (11.5, 36.9) | *<0.001* | -11.6 | (-23.4, 0.2) | *0.055* | -12.6 | (-19.5, -5.7) | *<0.001* |
| 65-69 | -4.0 | (-17.1, 9.0) | *0.544* | 5.6 | (-7.4, 18.7) | *0.395* | -1.6 | (-10.1, 6.9) | *0.709* |
| 70-74 | 12.2 | (-2.0, 26.3) | *0.092* | -3.7 | (-17.8, 10.3) | *0.600* | -8.4 | (-15.6, -1.2) | *0.022* |
| 75-79 | 6.7 | (-7.0, 20.4) | *0.337* | 1.4 | (-10.8, 13.7) | *0.819* | -8.1 | (-16.5, 0.4) | *0.061* |
| 80+ | Ref. | | | Ref. | | | Ref. | | |
| **Sex** |  |  |  |  |  |  |  |  |  |
| Female | Ref. | | | Ref. | | | Ref. | | |
| Male | 2.3 | (-6.8, 11.4) | *0.621* | 1.9 | (-5.2, 9.0) | *0.596* | -4.2 | (-10.0, 1.6) | *0.158* |
| **Urban/Rural** |  |  |  |  |  |  |  |  |  |
| Rural | Ref. | | | Ref. | | | Ref. | | |
| Urban | -8.0 | (-20.5, 4.5) | *0.207* | 11.1 | (-0.7, 22.9) | *0.065* | -3.1 | (-10.6, 4.4) | *0.413* |
| **Living Arrangement** |  |  |  |  |  |  |  |  |  |
| Living alone | 4.6 | (-5.2, 14.5) | *0.356* | -7.6 | (-19.5, 4.2) | *0.206* | 3.0 | (-7.6, 13.7) | *0.578* |
| Living with spouse only | -8.3 | (-26.6, 10.0) | *0.373* | 0.5 | (-14.4, 15.3) | *0.951* | 7.8 | (-8.1, 23.7) | *0.332* |
| Living with children | -7.7 | (-18.9, 3.5) | *0.176* | 8.2 | (-1.7, 18.0) | *0.104* | -0.5 | (-7.1, 6.1) | *0.892* |
| Other types of arrangement | Ref. | | | Ref. | | | Ref. | | |
| **Employment** |  |  |  |  |  |  |  |  |  |
| Not working | Ref. | | | Ref. | | | Ref. | | |
| Working | -9.1 | (-18.4, 0.2) | *0.056* | -1.7 | (-9.9, 6.5) | *0.684* | 10.8 | (3.8, 17.8) | *0.003* |
| **Health Insurance^b^** |  |  |  |  |  |  |  |  |  |
| No health insurance | Ref. | | | Ref. | | | Ref. | | |
| Has health insurance | -5.9 | (-15.3, 3.5) | *0.217* | 5.0 | (-5.3, 15.3) | *0.340* | 0.9 | (-5.4, 7.2) | *0.776* |
| *Note*. Sample restricted to those classified as *hypertensive* and not classified as *diagnosed,* both as defined in **Figure 1**. Estimates are averaged, adjusted, absolute risk differences between each group and the reference group obtained from a multinomial probit model of health facility visited (none, public or private) as a function of the wealth group indicators, all the covariates in Table 3, plus city/province indicators. pp = percentage point difference. Ref. = reference group. *P* is the P-value for the test of no difference from the reference group. | | | | | | | | | |

| **Table S5**. Prevalence of hypertensive crisis (SBP ≥ 180 or DBP ≥ 120) and percentages of those in crisis diagnosed and with a missed opportunity for diagnosis, older (≥ 60 years) adults, Philippines 2018/19 | | | | | | |
| --- | --- | --- | --- | --- | --- | --- |
|  | Hypertensive crisis prevalence  (n = 3861) | | In hypertensive crisis (n = 551) | | | |
|  |  |  | Diagnosed | | Missed opportunity | |
|  | % | (95% CI) | % | (95% CI) | % | (95% CI) |
| **Overall** | 8.4 | (6.7, 10.4) | 67.3 | (57.8, 75.9) | 1.1 | (0.6, 2.0) |
|  |  | |  | |  | |
| **Wealth** *P* | *0.340* | | *0.075* | | *0.085* | |
| Poorest | 10.0 | (5.6, 16.4) | 56.9 | (42.1, 70.7) | 0.6 | (0.1, 2.2) |
| Poorer | 9.1 | (6.0, 13.3) | 54.1 | (32.9, 74.2) | 1.0 | (0.2, 4.1) |
| Middle | 9.8 | (5.2, 16.7) | 76.3 | (59.8, 88.2) | 0.8 | (0.2, 3.1) |
| Richer | 5.8 | (3.6, 9.0) | 69.9 | (52.2, 83.8) | 3.5 | (1.4, 7.8) |
| Richest | 7.5 | (5.1, 10.5) | 82.8 | (63.4, 93.9) | 0.5 | (0.1, 2.2) |
| **Education** *P* | *<0.001* | | *0.057* | | *0.371* | |
| Elementary or less | 8.0 | (6.0, 10.5) | 61.7 | (51.3, 71.3) | 1.3 | (0.6, 2.6) |
| High school | 12.5 | (7.6, 19.2) | 81.1 | (64.2, 92.0) | 0.6 | (0.1, 2.2) |
| College | 2.2 | (1.3, 3.7) | 80.3 | (59.7, 92.7) | - |  |
| **Age, years** *P* | *0.041* | | *0.462* | | *<0.001* | |
| 60-64 | 5.9 | (3.7, 9.0) | 79.5 | (62.3, 90.9) | 0.3 | (0.0, 1.6) |
| 65-69 | 9.6 | (6.0, 14.5) | 67.4 | (45.0, 84.9) | 0.9 | (0.2, 2.9) |
| 70-74 | 8.3 | (6.2, 11.0) | 62.0 | (45.9, 76.2) | 4.5 | (1.7, 10.3) |
| 75-79 | 14.0 | (9.6, 19.7) | 60.0 | (36.8, 80.1) | 0.2 | (0.0, 1.0) |
| 80+ | 11.1 | (8.0, 14.9) | 57.5 | (39.4, 74.2) | 10.3 | (4.7, 19.6) |
| **Sex** *P* | *0.507* | | *0.560* | | *0.918* | |
| Female | 8.9 | (6.8, 11.3) | 69.5 | (57.2, 79.9) | 1.1 | (0.5, 2.3) |
| Male | 7.9 | (5.7, 10.7) | 63.5 | (46.4, 78.2) | 1.0 | (0.4, 2.6) |
| **Location** *P* | *0.728* | | *0.047* | | *0.010* | |
| Rural | 8.7 | (6.6, 11.2) | 60.7 | (48.7, 71.8) | 0.4 | (0.1, 1.6) |
| Urban | 8.1 | (5.6, 11.4) | 77.2 | (65.0, 86.6) | 2.3 | (1.3, 3.7) |
| **Living** *P* | *0.026* | | *0.562* | | *0.225* | |
| Alone | 11.9 | (6.8, 19.2) | 70.8 | (48.0, 87.4) | 0.2 | (0.0, 2.3) |
| With spouse only | 15.0 | (8.3, 24.7) | 64.7 | (35.8, 86.8) | 1.3 | (0.2, 5.8) |
| With children | 6.6 | (5.1, 8.5) | 63.9 | (53.2, 73.5) | 0.8 | (0.3, 2.0) |
| Other | 8.6 | (5.0, 14.0) | 75.5 | (57.9, 88.1) | 2.8 | (1.0, 6.9) |
| **Employment** *P* | *0.282* | | *0.676* | | *0.045* | |
| Not working | 9.3 | (7.4, 11.6) | 65.7 | (53.2, 76.7) | 1.6 | (0.8, 3.0) |
| Working | 7.4 | (5.0, 10.7) | 70.1 | (53.2, 83.5) | 0.3 | (0.0, 1.2) |
| **Health Insurance** | *0.809* | | *0.926* | | *0.473* | |
| No | 8.8 | (5.5, 13.3) | 68.0 | (51.2, 81.7) | 0.7 | (0.1, 2.6) |
| Yes | 8.4 | (6.8, 10.2) | 67.2 | (56.4, 76.7) | 1.2 | (0.6, 2.1) |
| *Note*. SBP = systolic blood pressure, DBP = diastolic blood pressure. *Diagnosed* and *Missing Opportunity* (for diagnosis) as defined in **Figure 1**. Percentages by covariates were adjusted for age and sex. *P* (values) for chi-squared of equal percentages across categories of the respective covariate. | | | | | | |

**Supplementary Checklist S1.** STROBE Statement for cross-sectional study

|  | **Item No** | **Recommendation** | **Page No.** | **Relevant text from manuscript** | |
| --- | --- | --- | --- | --- | --- |
| **Title and abstract** | 1 | (*a*) Indicate the study’s design with a commonly used term in the title or the abstract | 1 | *cross-sectional analysis of nationally representative individual-level data* | |
|  |  | (*b*) Provide in the abstract an informative and balanced summary of what was done and what was found | 2 | *Methods* and *Findings* sections of *Abstract* | |
| **Introduction** | | |  | |  |
| Background/rationale | 2 | Explain the scientific background and rationale for the investigation being reported | 3-4 | Text of *Introduction* | |
| Objectives | 3 | State specific objectives, including any prespecified hypotheses | 4 | *This study aimed to quantify the extent to which effective implementation of opportunistic screening at all health facilities in the Philippines would increase the diagnosis of hypertension and change socioeconomic inequality in the rate of diagnosis in the older population.* | |
| **Methods** | | |  | |  |
| Study design | 4 | Present key elements of study design early in the paper | 1-2, 4-7 | *Title* and *Methods* section of *Abstract.*  *Methods, Data*, *Measurements and Outcomes*, *Covariates,* *Statistical Analysis.* | |
| Setting | 5 | Describe the setting, locations, and relevant dates, including periods of recruitment, exposure, follow-up, and data collection | 4-5 | *We used data from the baseline of the Longitudinal Study of Ageing and Health in the Philippines (LSAHP), conducted from October 2018 to February 2019.* | |
| Participants | 6 | (*a*) Give the eligibility criteria, and the sources and methods of selection of participants | 4-6 | *a nationally representative sample of Filipinos aged 60 years and older (60+)*  *We restricted the analysis sample to respondents with three BP measurements* | |
| Variables | 7 | Clearly define all outcomes, exposures, predictors, potential confounders, and effect modifiers. Give diagnostic criteria, if applicable | 5-6 | *Methods: Measurements and Outcomes, Covariates* | |
| Data sources/ measurement | 8* | For each variable of interest, give sources of data and details of methods of assessment (measurement). Describe comparability of assessment methods if there is more than one group | 5-6 | *Methods: Measurements and Outcomes, Covariates* | |
| Bias | 9 | Describe any efforts to address potential sources of bias | 7-8 | *We estimated … with adjustment for age and sex.*  *We estimated fully adjusted differences …*  *We applied sampling weights in all analyses ..* | |
| Study size | 10 | Explain how the study size was arrived at | 24 | *Figure 1*  As is common for a multi-purpose survey, the sample size was not determined by a power calculation for estimation of a particular parameter. Allowing for expected non-response, the target sample was set to 6,335 with the intention of achieving a sample of 6,000.^1^ | |
| Quantitative variables | 11 | Explain how quantitative variables were handled in the analyses. If applicable, describe which groupings were chosen and why | 5-6 | *Methods: Measurements and Outcomes, Covariates* | |
| Statistical methods | 12 | (*a*) Describe all statistical methods, including those used to control for confounding | *7-8* | *We estimated … with adjustment for age and sex. Adjustment was done by …*  *We estimated fully adjusted differences … by estimating …* | |
|  |  | (*b*) Describe any methods used to examine subgroups and interactions | *N/A* | No analysis of subgroups or interaction. | |
|  |  | (*c*) Explain how missing data were addressed | 6  17 | *We restricted the analysis sample to respondents with three BP measurements … There were no missing data on wealth and covariates.*  *The analysis sample excluded 405 respondents whose blood pressure could not be measured …* | |
|  |  | (*d*) If applicable, describe analytical methods taking account of sampling strategy | 6-7 | *Statistical Analysis* | |
|  |  | (*e*) Describe any sensitivity analyses | SM | Table S3. Missed opportunities at clinics and hospitals. | |
| **Results** | | |  | |  |
| Participants | 13* | (a) Report numbers of individuals at each stage of study—eg numbers potentially eligible, examined for eligibility, confirmed eligible, included in the study, completing follow-up, and analysed | 24 | *Figure 1* | |
|  |  | (b) Give reasons for non-participation at each stage | 24 | *Figure 1* | |
|  |  | (c) Consider use of a flow diagram | 24 | *Figure 1* | |
| Descriptive data | 14* | (a) Give characteristics of study participants (eg demographic, clinical, social) and information on exposures and potential confounders | 25 | *Table 1* | |
|  |  | (b) Indicate number of participants with missing data for each variable of interest | 24 | *Figure 1* | |
| Outcome data | 15* | Report numbers of outcome events or summary measures | *26* | Table 2 | |
| Main results | 16 | (*a*) Give unadjusted estimates and, if applicable, confounder-adjusted estimates and their precision (eg, 95% confidence interval). Make clear which confounders were adjusted for and why they were included | *26-28* | *Tables 2-4* | |
|  |  | (*b*) Report category boundaries when continuous variables were categorized | 25 | *Table 1* | |
|  |  | (*c*) If relevant, consider translating estimates of relative risk into absolute risk for a meaningful time period | 27-28 | *Tables 3 & 4* | |
| Other analyses | 17 | Report other analyses done—eg analyses of subgroups and interactions, and sensitivity analyses | SM | *Tables S2, S3, S5* | |
| **Discussion** | | |  | |  |
| Key results | 18 | Summarise key results with reference to study objectives | *11*  *12*  *12*  *14* | *we found high (around two thirds) prevalence of hypertension and low diagnosis of the condition in the older (60+) population of the Philippines.*  *Almost two fifths of those classified as hypertensive remained undiagnosed …*  *We found that this screening could increase the rate of diagnosis of the older, hypertensive population of the Philippines by almost twelve percentage points.*  *The increase in diagnosis would, however, be smallest – absolutely and relatively – among the poorest. Effective opportunistic screening would exacerbate socioeconomic inequality in the diagnosis of hypertension* | |
| Limitations | 19 | Discuss limitations of the study, taking into account sources of potential bias or imprecision. Discuss both direction and magnitude of any potential bias | *15-17* | *Strengths and limitations* | |
| Interpretation | 20 | Give a cautious overall interpretation of results considering objectives, limitations, multiplicity of analyses, results from similar studies, and other relevant evidence | 17 | *This study demonstrated that opportunistically screening older Filipinos for hypertension at health facilities has strong potential to increase diagnosis … our estimates suggest that, … opportunistic screening would exacerbate inequality in the diagnosis of hypertension* | |
| Generalisability | 21 | Discuss the generalisability (external validity) of the study results | 12 | *Health system and policy implications* | |
| **Other information** | | |  | |  |
| Funding | 22 | Give the source of funding and the role of the funders for the present study and, if applicable, for the original study on which the present article is based | *8,18* | *Role of Funding Source*,  *Funding Source* | |

**References**

1. Barrios EB and Marquez MPN. Annex A: LSAHP Sampling Design and Weights, in G. T. Cruz, C. J. P. Cruz, and Y. Saito (eds.), Ageing and Health in the Philippines, pp. 227-234, 2019. Jakarta, Indonesia: Economic Research Institute for ASEAN and East Asia. <https://www.eria.org/uploads/media/Books/2019-Dec-Ageing-and-Health-Philippines/21-Ageing-and-Health-Philippines-Annexes-new.pdf> (accessed July 19, 2024).
